# Dithiophosphate-Induced Redox Conversions of Reduced and Oxidized Glutathione

**DOI:** 10.3390/molecules26102973

**Published:** 2021-05-17

**Authors:** Rezeda A. Ishkaeva, Ilyas S. Nizamov, Dmitriy S. Blokhin, Elizaveta A. Urakova, Vladimir V. Klochkov, Ilnar D. Nizamov, Bulat I. Gareev, Diana V. Salakhieva, Timur I. Abdullin

**Affiliations:** 1 Department of Biochemistry, Biotechnology, and Pharmacology, Institute of Fundamental Medicine and Biology, Kazan Federal University, 420008 Kazan, Russia; RezAAhmadishina@kpfu.ru (R.A.I.); Dmitrij.Blohin@kpfu.ru (D.S.B.); DiVitSai@gmail.com (D.V.S.); 2 Department of High Molecular and Organoelement Compounds, Alexander Butlerov Institute of Chemistry, Kazan Federal University, 420008 Kazan, Russia; Ilyas.Nizamov@kpfu.ru (I.S.N.); IDNizamov@kpfu.ru (I.D.N.); 3 Kazan Scientific Center, Laboratory of Organometallic and Coordination Compounds, A.E. Arbuzov Institute of Organic and Physical Chemistry, Russian Academy of Sciences, 420088 Kazan, Russia; 4 Department of Medical Physics, Institute of Physics, Kazan Federal University, 420008 Kazan, Russia; urakova.li@yandex.ru (E.A.U.); Vladimir.Klochkov@kpfu.ru (V.V.K.); 5 Laboratory of Isotope and Element Analysis, Institute of Geology and Petroleum Technologies, Kazan Federal University, 420008 Kazan, Russia; bulat@gareev.net

**Keywords:** glutathione, dithiophosphates, redox properties, hydrolytic properties, hydrogen sulfide, thiol, disulfide

## Abstract

Phosphorus species are potent modulators of physicochemical and bioactive properties of peptide compounds. O,O-diorganyl dithiophoshoric acids (DTP) form bioactive salts with nitrogen-containing biomolecules; however, their potential as a peptide modifier is poorly known. We synthesized amphiphilic ammonium salts of O,O-dimenthyl DTP with glutathione, a vital tripeptide with antioxidant, protective and regulatory functions. DTP moiety imparted radical scavenging activity to oxidized glutathione (GSSG), modulated the activity of reduced glutathione (GSH) and profoundly improved adsorption and electrooxidation of both glutathione salts on graphene oxide modified electrode. According to NMR spectroscopy and GC–MS, the dithiophosphates persisted against immediate dissociation in an aqueous solution accompanied by hydrolysis of DTP moiety into phosphoric acid, menthol and hydrogen sulfide as well as in situ thiol-disulfide conversions in peptide moieties due to the oxidation of GSH and reduction of GSSG. The thiol content available in dissolved GSH dithiophosphate was more stable during air oxidation compared with free GSH. GSH and the dithiophosphates, unlike DTP, caused a thiol-dependent reduction of MTS tetrazolium salt. The results for the first time suggest O,O-dimenthyl DTP as a redox modifier for glutathione, which releases hydrogen sulfide and induces biorelevant redox conversions of thiol/disulfide groups.

## 1. Introduction

Glutathione tripeptide (L-γ-glutamyl-L-cysteinyl-glycine, GSH) is the predominant antioxidant in mammalian cells; its concentration in cellular compartments is generally ≤ 10 mM [1,2]. Cellular requirements for GSH are covered by intracellular synthesis, in which the linking of Gly residue to Glu-Cys dipeptide is the rate-limiting step [3]. The antioxidant activity of GSH covers the elimination of reactive oxygen species (ROS), such as hydrogen peroxide, hydroxyl radical and lipid peroxyl radicals, at the expense of the thiol group of Cys residue. These reactions occur non-enzymatically and are catalyzed by enzymes, such as glutathione peroxidase, which produces the oxidized glutathione (GSSG) further reduced by NADPH-dependent glutathione reductase [4]. Another important function of GSH is cell detoxification via the formation of S-adducts with harmful electrophilic compounds by glutathione-S-transferase, as well as the elimination of cytotoxic α-oxoaldehydes by glyoxalases I and II [5].

Most GSH-dependent enzymes have proved to be pharmacological targets for anticancer [6,7] and antiparasitic [8,9,10] agents such as GSH derivatives and mimetics with enzyme inhibitory activity. The redox properties of GSH *per se* have attracted great interest in the therapy of degenerative, inflammatory, immunological and viral diseases accompanied by oxidative stress and thiol depletion in cells [3,11].

However, poor pharmacokinetic characteristics of free GSH restrict its therapeutic potential [12]. A common strategy to solve the problem relies on the covalent modification of the tripeptide with lipophilic groups to increase its bioavailability and stability. Different GSH modifications have been proposed to date, e.g., GSH monoethylester, S-acetylglutathione, N-butanoyl GSH [1], S-trityl-L-cysteine derivatives [13] and N-glutathione conjugates with alkyl chains [14]. These compounds were generally designed as antiviral agents to decrease virus replication and/or provide immunomodulatory effects [1,15]. In addition, GSH conjugates with a fatty acid, namely, S-lauroylglutathione and S-palmitoleoylglutathione, were shown to protect SH-SY5Y cells and cholinergic neurons against amyloid-induced oxidative damage and apoptosis [16,17].

Alternatively, the formation of non-covalent complexes of GSH is of interest to modulate properties of the tripeptide without modification of its intact structure. The reported complexes of GSH mainly relate to coordination compounds with transition metals [18,19,20,21,22]. The complexation with GSH plays important biological roles, including deposition, modulation and transportation of the metal ions. It also potentially allows the regulation of zwitterionic and redox properties of GSH [18,19,20,21]; however, the reactivity and potential toxicity of transition metals restrict their use in GSH formulations.

We have shown that dithiophosphoric acid (DTP) with O,O-diorganyl groups can protonate nitrogen-containing biomolecules with the participation of the acidic thiol group of DTP. The reaction produces ammonium salts between biomolecule (cation) and DTP (anion) with new properties and bioactivity over the constituents [23,24]. The potential of the DTP modifier in the regulation of biospecific properties of GSH and other peptide molecules is poorly known. This work focuses on the examination of redox and hydrolytic behaviors of GSH dithiophosphates in relation to their expected therapeutic applications. O,O-di-(–)-menthyl DTP with pharmaceutically relevant lipophilic l-menthyl groups was used to modify glutathione in reduced and oxidized forms.

## 2. Results and Discussion

### 2.1. Redox Properties of Glutathione Dithiophosphates

#### 2.1.1. Structure Characterization

**Figure 1:**
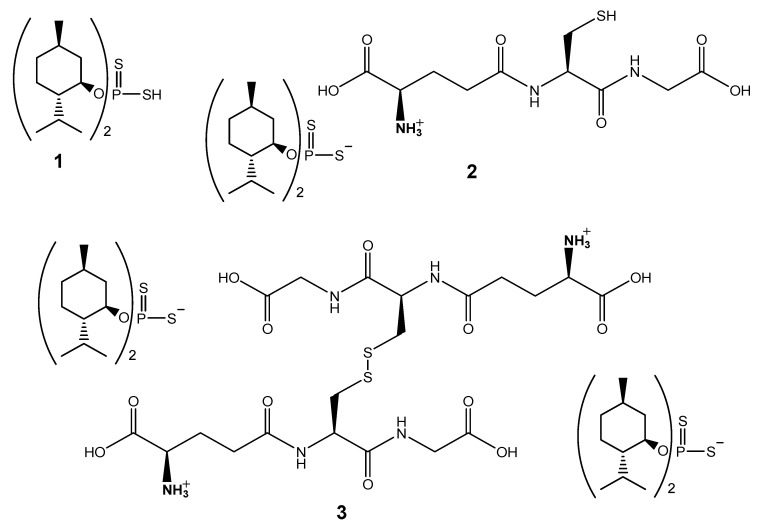
Structural formulas of O,O-(–)-dimenthyl dithiophoshoric acid (**1**) and ammonium salts of **1** with reduced glutathione (**2**) and oxidized glutathione (**3**).

The pre-synthesized O,O-(–)-dimenthyl dithiophoshoric acid (DTP, **1**) was reacted with GSH or GSSG as detailed in Supplementary Materials. The coupling reaction of DTP with the tripeptide is based on proton transfer from the ionizable thiol group of DTP to the α-amino group of Glu residue to form an ionic bond between the moieties. The structure of synthesized ammonium salts GSH-DTP (**2**) and GSSG-(DTP)_2_ (**3**) (Figure 1) was confirmed by NMR, FTIR and elemental analyses; no racemization of the peptide moieties occurred according to polarimetry measurements (Supplementary Materials).

The glutathione dithiophosphates, unlike the initial peptides, were well-soluble in polar organic solvents such as DMSO and alcohols, which are compatible with biological assays. Considering DMSO-promoted oxidation of GSH [25], methanol was preferred to prepare stock solutions of the salts. The compounds were sufficiently water-soluble at submillimolar concentrations; at millimolar concentrations, their solubilization was assisted by detergents. Such solvent requirements indicate amphiphilic properties of the glutathione dithiophosphates. The compounds were not compatible with pyrene fluorescence assay to determine their critical micelle concentration, apparently due to the quenching effect of the menthol component [26].

**Figure 2:**
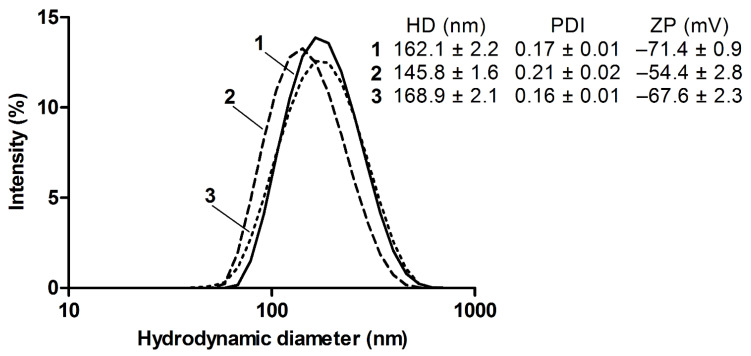
The distribution of hydrodynamic diameter of associates of the compounds **1**, **2**, and **3** (1 mM in HEPES buffer, pH = 7.0). Inset: Mean ± SD (*n* = 3) values of hydrodynamic diameter, polydispersity index (PDI), and zeta potential.

Dynamic light scattering confirmed that the compounds formed homogeneous aggregates in an aqueous solution with a hydrodynamic diameter of up to ca. 170 nm (Figure 2) attributed to menthyl group-mediated hydrophobic association of DTP moiety. Zeta potential values (mV) of the aggregates measured in HEPES buffer (pH = 7) were as follows: –71.4 (**1**), –54.4 (**2**) and –67.6 (**3**), apparently reflecting the acidic properties of the aggregate–solution interface (given that the thiol group of DTP is more acidic than the carboxyl groups of GSH).

#### 2.1.2. Radical Scavenging Activity

**Figure 3:**
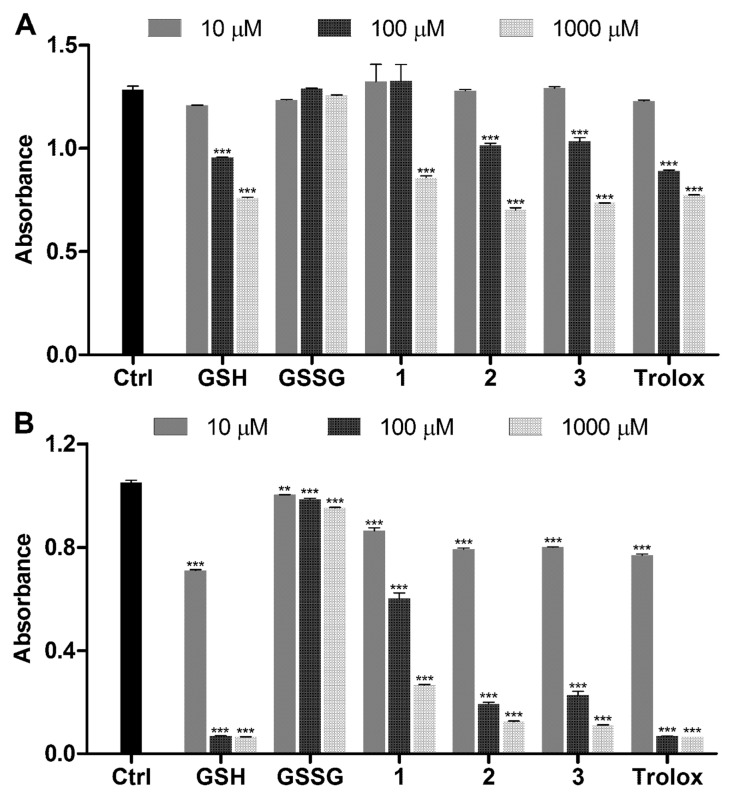
Radical scavenging activity of glutathione dithiophosphates in PBS: (**A**) DPPH assay and (**B**) ABTS assay. Concentration of the probes is 0.25 mM. Mean ± SD (*n* = 3, ** *p* < 0.01, *** *p* < 0.001 vs. Ctrl) are shown; Ctrl is the signal of probes alone.

The glutathione dithiophosphates were assessed using DPPH and ABTS radical scavenging assays in PBS (Figure 3). For comparison purposes, the concentrations of dimeric compounds, namely, GSSG and **3** were presented per one GSH/DTP unit.

GSH, unlike GSSG, caused the concentration-dependent inactivation of DPPH (Figure 3A) and ABTS (Figure 3B) radicals due to the scavenging effect of the thiol group. DTP alone possessed some antiradical activity attributed to the thiol group adjacent to the phosphorus atom. In comparison with GSH, the activity of GSH-DTP was similar in DPPH assay and somewhat lower in ABTS assay, suggesting a modulation effect of DTP moiety in GSH dithiophosphate. Interestingly, GSSG-(DTP)_2,_ in contrast to GSSG, exhibited antiradical activity comparable to that of GSH-DTP (Figure 3).

The results show that DTP *per se* has the ability to scavenge radicals, which is inferior to that of GSH. In dithiophosphates, however, DTP activity is manifested differently depending on the redox state of glutathione. DTP moiety imparts a profound scavenging effect to GSSG-(DTP)_2_ while does not provide any additive effect in GSH-DTP in comparison with the unmodified peptides. This could be explained by an internal redox reaction between DTP and peptide moieties of the dithiophosphates in an aqueous environment (as considered in subsequent sections).

#### 2.1.3. Electrochemical Behavior

**Figure 4:**
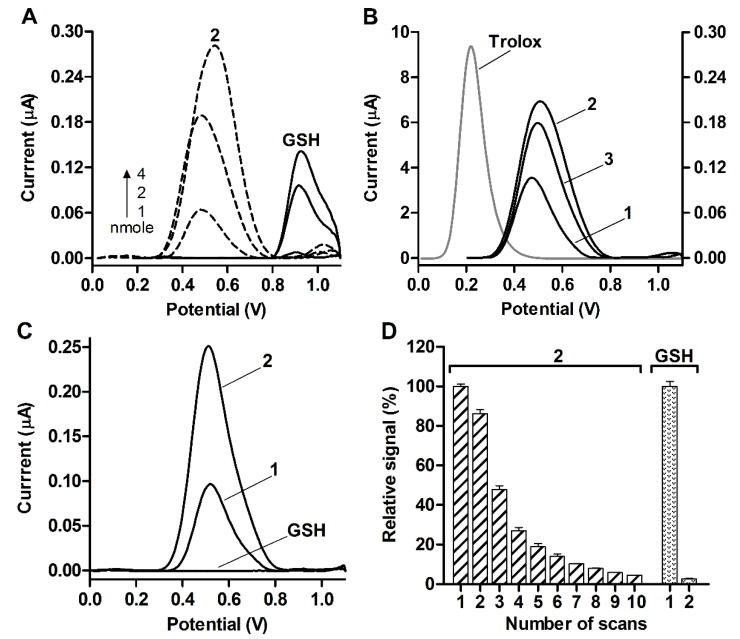
Voltammetric behavior of glutathione, DTP (**1**), GSH-DTP (**2**) and GSSG-(DTP)_2_ (**3**) on GOE. Square-wave voltammograms of (**A**) GSH and **2** air-dried at different loading; (**B**) **1**, **2**, **3** (right Y-axis) and Trolox (left Y-axis) air-dried at a 4 nmole loading; (**C**) **1** and **2** pre-adsorbed from 500 µM solution in PBS (without drying); (**D**) relative signals of air-dried GSH and **2** upon repetitive scanning (vs. the first signal = 100%). SWV parameters: frequency 10 Hz, amplitude 10 mV, potential step 5 mV.

Electrochemical techniques are an important tool for studying redox properties and conversions of drug molecules [27]. The glutathione dithiophosphates were analyzed by square-wave voltammetry using a graphene oxide modified electrode (GOE). GSH was oxidized at a potential of ca. 0.9 V vs. Ag/AgCl in proportion to its surface loading (Figure 4A). No peaks were produced by GSSG on GOE as well as GSH on unmodified glassy carbon electrode, respectively, confirming that the electrooxidation of GSH involves the thiol group of Cys residue and is mediated by graphene oxide modifier. It still occurred at an overpotential, indicating poor electrochemical behavior of GSH (Figure 4A). Previously, carbon nanomaterial-assisted electrooxidation of GSH was observed at different potentials [28,29,30,31].

GSH-DTP, GSSG-(DTP)_2_ as well as DTP were oxidized on GOE at considerably lower and close potentials around 0.5 V and generated increased currents compared with GSH (Figure 4). These data show that DTP has its own electroactivity with its thiol group as an expected redox center. Furthermore, DTP moiety seems to determine the oxidation of both dithiophosphates. Nevertheless, the peak current of GSH-DTP was significantly higher than that of DTP, namely 0.21 ± 0.02 µA vs. 0.15 ± 0.06 µA (*p* < 0.05), for the same surface loading (4 nmole per electrode), indicating the contribution of peptide groups to the signal of dithiophosphate.

The symmetric shape of peaks generated by the compounds **1**, **2**, and **3** (Figure 4A, B) was typical of electrochemical reactions of strongly adsorbed species. This suggests that DTP also provides effective adsorption of the dithiophosphates on GOE, presumably due to the hydrophobic association of the menthyl groups with graphene oxide sheets. This adsorption occurred even upon brief incubation of GOE with the dithiophosphate solution but not the GSH solution (Figure 4C), which should be dried on GOE to detect GSH (Figure 4A). Moreover, surface-attached GSH-DTP was characterized by gradual oxidation at GOE during ca. 10 scans, whereas all GSH molecules at the same loading were oxidized during a single scan (Figure 4D), further supporting the enhanced electrochemical activity of the GSH derivative.

These results show that the glutathione dithiophosphates exhibit profoundly improved electrochemical adsorption and oxidation mediated by DTP moiety. DTP provides similar electrooxidation efficiency for the dithiophosphates of GSH and GSSG (Figure 4B) in accordance with their similar ability to scavenge radicals (Figure 3), together suggesting both compounds as potential antioxidants. Furthermore, DTP-mediated adsorption could be useful to immobilize glutathione molecules on materials surfaces and potentially probe glutathione-targeted biochemical reactions using dithiophosphate modified electrodes.

#### 2.1.4. Air Oxidation

**Figure 5:**
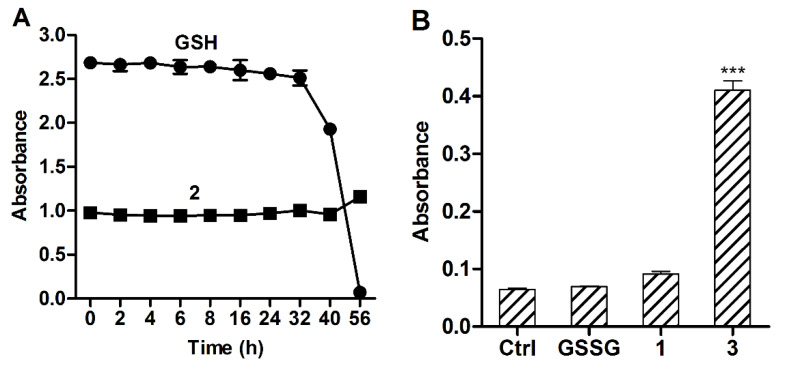
Analysis of thiol content in glutathione dithiophosphates in PBS (pH = 7.4) using the Ellman method. (**A**) Kinetics of air oxidation of GSH and GSH-DTP (**2**) upon stirring. (**B**) Control signal of as dissolved GSSG, DTP (**1**), and GSSG-(DTP)_2_ (**3**). The concentration of the compounds and DTNB was 0.5 mM. Mean ± SD (*n* = 3, *** *p* < 0.001) are shown.

Given the susceptibility of GSH to oxidation by atmospheric oxygen, the stability of thiol groups of GSH and GSH-DTP in PBS was assessed by the Ellman assay (Figure 5A). Control measurements showed that DTP weakly reacted with DTNB, whereas GSSG-(DTP)_2_, in great contrast to GSSG, unexpectedly provided a noticeable signal (Figure 5B). On the contrary, the as-dissolved GSH-DTP exhibited decreased reactivity to DTNB compared with GSH (Figure 5A). These findings could be explained by in situ redox reactions of GSSG-(DTP)_2_ and GSH-DTP in an aqueous solution to cause, respectively, the appearance or depletion of thiol groups in the peptide moieties.

A weak decay of the thiol content in GSH solution was observed during 32 h incubation, which was followed by sharp and complete oxidation of the tripeptide to 56 h. Interestingly, no thiol oxidation was detected in the case of GSH-DTP during this time period (Figure 4A). Therefore, in spite of the initial depletion of thiol groups, the dithiophosphate shows decreased susceptibility to be further oxidized at ambient conditions.

#### 2.1.5. Interaction with Tetrazolium Probe

**Figure 6:**
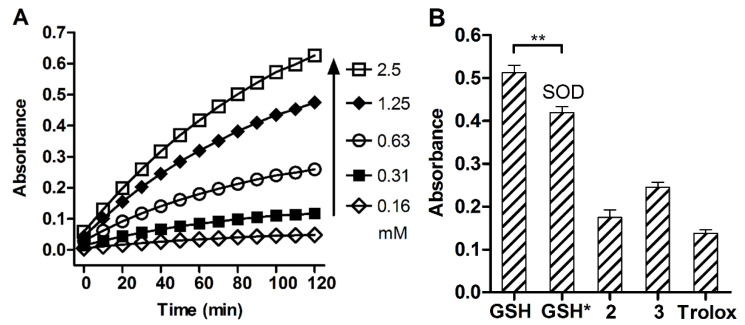
The reductive ability of glutathione dithiophosphates toward MTS probe in PBS (pH = 7.4). (**A**) Concentration and time dependent effect of GSH. (**B**) Effect of GSH, GSH + SOD (GSH*), GSH-DTP (**2**), GSSG-(DTP)_2_ (**3**) and Trolox. Concentration of the reductants is 1 mM, SOD activity is 500 U/mL; incubation time is 2 h. Mean ± SD are shown (*n* = 3, ** *p* < 0.01).

A water-soluble tetrazolium salt MTS was used as a versatile probe for reductive species, including the superoxide radical generated upon one-electron reduction of O_2_ [32]. GSH was found to reduce MTS in proportion to the tripeptide concentration (Figure 6A). The reaction was partially inhibited by superoxide dismutase (Figure 6B, *p* < 0.05), indicating GSH-induced formation of superoxide radical, which is also capable of reduction of tetrazolium salts [32]. Earlier, superoxide radical generation by GSH in complex with copper was reported [33].

GSSG and DTP did not react with the probe, indicating the role of Cys thiol in the reaction. Compared with GSH, the dithiophosphates had a weaker reductive ability toward MTS (Figure 6B) attributed to available thiol groups of the peptide moiety rather than the inhibitory effect of DTP moiety. These results agree with the Ellman assay (Figure 5), together suggesting some in situ conversions of thiol and disulfide groups of the glutathione dithiophosphates in an aqueous solution. To explain such redox behavior of the compounds, their hydrolytic stability and thiol-disulfide conversions were analyzed by NMR spectroscopy and GC–MS.

### 2.2. Hydrolytic Behavior of Glutathione Dithiophosphates

#### 2.2.1. NMR Analysis

According to 1D ^31^P{^1^H} NMR analysis in the H_2_O–D_2_O system, the initial signal of DTP at 81.9 ppm rapidly disappeared along with the appearance of a new signal at 0 ppm, which corresponds to orthophosphoric acid (OPA) [34]. This shows that DTP alone undergoes complete hydrolysis upon which the phosphorus component is converted into OPA. The spectra of glutathione dithiophosphates with integral intensities are provided in Figures S1–S5. In unbuffered solution, GSH-DTP appeared in the form of initial undissociated salt **2** (109 ppm—31.5%) and OPA (0 ppm—68.5%), indicating considerable dissociation of **2** accompanied by the hydrolysis of free DTP anion (Figure S1, in Supplementary Materials).

In the presence of dodecylphosphocholine (DPC) used to assist structure characterization of peptides [35,36], GSH-DTP generated the signals of **2** (107.5 ppm—41%) and OPA (0 ppm—59%) (Figure S2). Apparently, DPC somewhat decreases the dissociation of GSH-DTP due to its hydrophobic association with the micelles. To assess GSH-DTP at physiological pH value, borate buffered saline (BBS, pH = 7.4) was used (Figure S4) since PBS interfered with the signal of OPA (Figure S3). In BBS, the salt **2** was much less dissociated (105.9 ppm—78.3%) against OPA (0 ppm—21.7%), and the spectra did not change during 3 days. This supports increased aqueous stability of GSH-DTP in BBS. In unbuffered solution, the hydrolysis of DTP moiety was accompanied by acidification, which seemingly further promotes the ionic dissociation of GSH-DTP. GSSG-(DTP)_2_ exhibited similar hydrolytic behavior (105.9 ppm—82.4%; 0 ppm—17.6%) (Figure S5).

**Figure 7:**
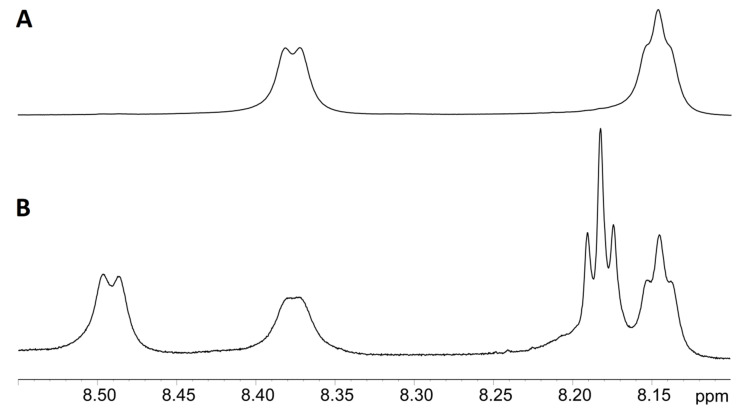
Fragments of 1D ^1^H NMR spectra of GSH (**A**) and GSH-DTP (**B**) at 25 °C in the BBS–D_2_O system (pH = 7.4).

In the ^1^H NMR spectrum of unmodified GSH, the NH protons of amide groups, namely, Cys HN and Gly HN, gave a doublet (8.37 ppm) and a triplet (8.13 ppm), respectively (Figure 7A). GSH-DTP was characterized by a split of the above signals of amide protons into two doublets at 8.39 and 8.51 ppm (NHCH) and two triplets at 8.48 and 8.44 ppm (NHCH_2_) (Figure 7B). No signal of NH_2_ α-amino group protons (Glu residue) was detected in GSH-DTP due to exchange coupling of the amino group with H_2_O/D_2_O protons. GSSG and GSSG-(DTP)_2_ provided a similar splitting pattern in the ^1^H NMR spectra (Figure S6) to that of GSH and GSH-DTP.

The β-carbon atom of a cysteine residue of GSH (Cys Cβ) was assigned to distinguish between the reduced and oxidized forms of glutathione. In 2D ^1^H-^13^C HSQC NMR spectra, the Cys Cβ signal shifted upon the disulfide bond formation from 25.6 ppm (GSH, Figure S7) to 38.7 ppm (Figure S8, GSSG). In addition, in the 1D ^1^H NMR spectra of GSSG Cys Hβ protons became nonequivalent and produced separate signals. The Cys Hβ protons of GSH resulted in two doublets at 3.0 ppm, whereas the analogous signals in GSSG were split into two pairs of doublets at 3.01 and 3.36 ppm (Figure S9).

**Figure 8:**
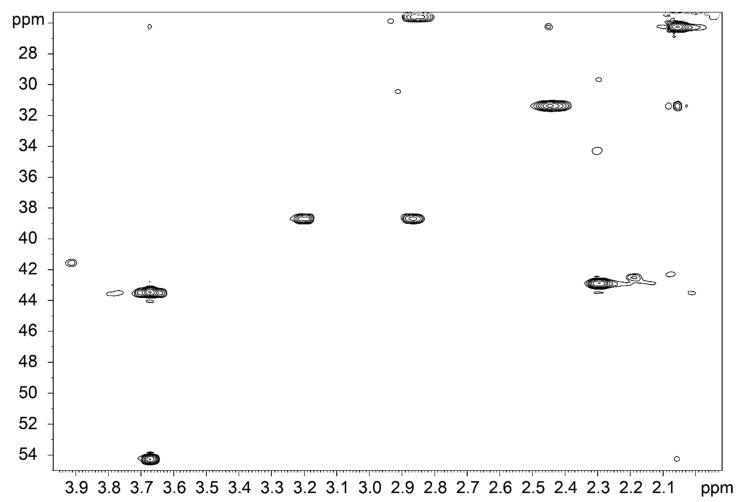
2D ^1^H-^13^C HSQC NMR spectrum of GSH-DTP in the BBS–D_2_O system at 25 °C (pH = 7.4).

In the ^1^H-^13^C HSQC NMR spectrum of GSH-DTP, both reduced (Cys Cβ 25.6 ppm—42%) and oxidized (Cys Cβ 38.7 ppm—58%) forms of the tripeptide were detected (Figure 8). Their ratio did not significantly change over time, at least for 3 days. Similarly, both redox forms of glutathione at the ratio of 1:1 appeared in the corresponding spectrum of GSSG-(DTP)_2_ (Figure S10). These data are in good agreement with probing with DTNB and MTS (Figure 5 and Figure 6), which showed the variation in thiol content of the glutathione derivatives.

Altogether, the NMR data demonstrate that the glutathione dithiophosphates, depending on the conditions, may exist in aqueous solutions in the undissociated form that appears as a tight ion pair, which gradually dissociates into the constituents. The released O,O-dimenthyl DTP undergoes hydrolysis accompanied by the conversion of thiol and disulfide groups in glutathione molecules. Such a redox reaction was attributed to a sulfur-containing species derived from the thiol/thione groups of DTP, presumably, hydrogen sulfide (H_2_S), since this product was detected upon the hydrolysis of dialkyldithiophosphoric acids [37].

#### 2.2.2. Detection of DTP-Derived H_2_S and Its Role in Glutathione Conversions

**Figure 9:**
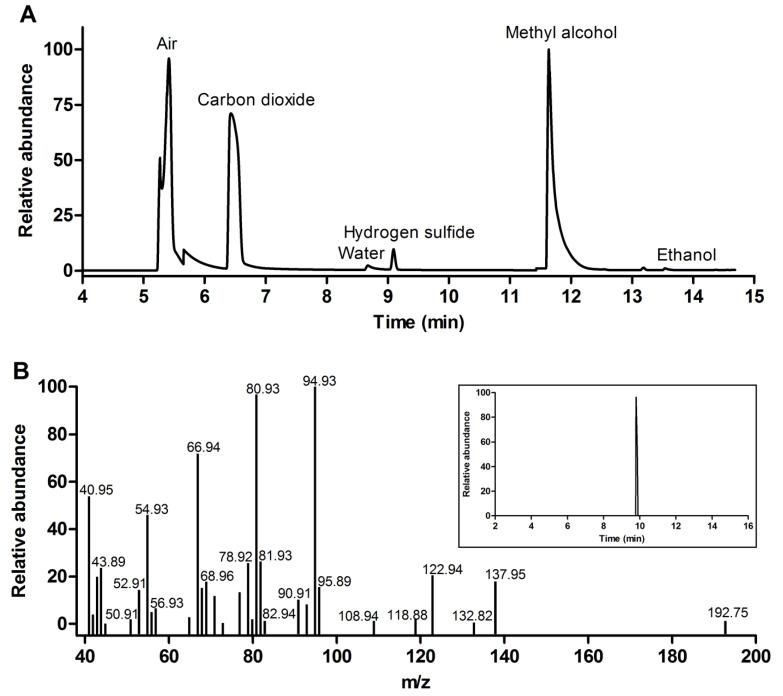
GC–MS analysis of hydrolysis products of GSH-DTP in methanol-water mixed solution: chromatogram of volatile products in the gas phase (**A**) and chromatogram (inset) and mass-spectrum of menthol in the liquid phase (**B**). See the Materials and Methods section for details.

To verify whether the hydrolysis of dithiophosphates was accompanied by H_2_S release, the compounds dissolved in methanol were mixed with water in a hermetic vial and subjected to GC–MS analysis. GSH-DTP generated a well-defined peak of H_2_S with a retention time of about 9.1 min found in the gas phase (Figure 9A) and liquid phase (Figure S11). Released H_2_S was also detected in DTP and GSSG-(DTP)_2_ samples in both phases (Figures S12–S15). In addition, the production of free menthol in dithiophosphate solutions was confirmed (Figure 9B). These data demonstrate that DTP is the donor of H_2_S both *per se* and in the form of glutathione dithiophosphates. The latter compounds can be considered a sustained release system for H_2_S, which is an established gasotransmitter with an important role in cancer and neurodegenerative diseases [38,39,40].

H_2_S is involved in complex condition-dependent and reversible interactions with endogenous disulfides and thiols [41]. It is known to reduce disulfides into corresponding thiols and persulfides [42]. Such a process explains well the appearance of thiol groups in GSSG-(DTP)_2_ in different analyses (Figure S10, Figure 5B and Figure 6B). Although H_2_S is not capable of direct interaction with reduced thiols, and hence with GSH and GSH-DTP, it may oxidize the tripeptide via a radical process involving thiyl radical [42] or other H_2_S-derived reactive sulfur species [43]. Interestingly, such species are generated upon H_2_S oxidation by superoxide radical [43].

**Figure 10:**
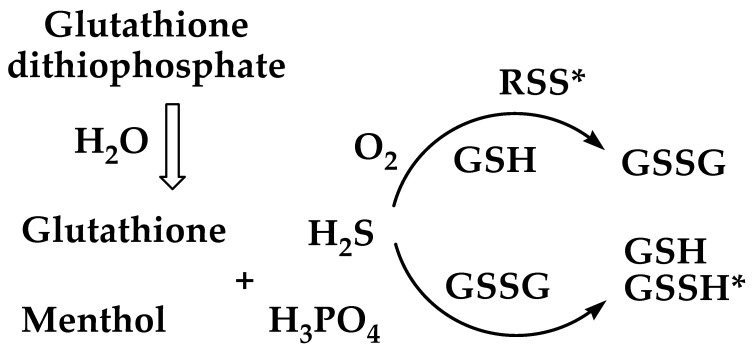
Proposed products of hydrolysis and in situ redox conversions of glutathione dithiophosphates. Expected labile intermediates are indicated by an asterisk: RSS—a reactive sulfur species, GSSH—glutathione persulfide.

These data, along with the evidence on superoxide radical formation in the presence of GSH at ambient conditions (Figure 6B), provide a rational explanation for the rapid conversion of thiol groups into disulfide groups in dissolved GSH-DTP (Figure 5 and Figure 8). Clarification of the mechanisms of GSH-DTP oxidation by a sulfur-containing radical in physiological conditions requires a separate study, which will be performed elsewhere. Nevertheless, based on our results, a general scheme for the hydrolytic and redox behavior of GSH-DTP and GSSG-(DTP)_2_ can be given (Figure 10).

The results of this study demonstrate that glutathione dithiophosphates exhibit specific redox properties in biologically relevant reactions. DTP moiety imparts enhanced radical scavenging and electrochemical activities to the dithiophosphates. In contrast to DTP alone, the dithiophosphates persist against immediate hydrolysis of DTP moiety. DTP-derived H_2_S release upon the hydrolysis mediates thiol-disulfide conversions depending on the redox form of glutathione. Depletion of thiol pool detected in dissolved GSH-DTP does not cause a considerable decrease of its antiradical activity (Figure 3), whereas the available thiol groups are more persistent to air oxidation compared with GSH (Figure 4B) attributed to the antioxidant effect of unhydrolyzed DTP moiety. In situ generation of H_2_S by the dithiophosphates and DTP-mediated redox conversions of reduced and oxidized glutathione (Figure 10) are of particular interest to develop redox-modulating prodrugs. Furthermore, DTP can be considered as a promising non-covalent modifier for other bioactive oligopeptides to alter their physicochemical and pharmaceutical properties.

## 3. Materials and Methods

### 3.1. Materials

Reduced glutathione, oxidized glutathione (purity 98%) and (–)-(1R,2S,5R)-menthol (purity 99.5%) were purchased from Acros Organics. 2,2′-diphenyl-1-picrylhydrazyl (DPPH), 2,2’-azino-bis(3-ethylbenzthiazoline-6-sulfonic acid) diammonium salt (ABTS), 5,5’-dithiobis-(2-nitrobenzoic acid) (DTNB), tetraphosphorus decasulfide (purity 99%), Triton X100 and superoxide dismutase (SOD) from bovine erythrocytes were purchased from Sigma-Aldrich. Single-layer graphene oxide (purity 99%) was produced by Cheap Tubes Inc. CellTiter 96^®^ Aqueous MTS Reagent Powder was purchased from Promega. Milli-Q grade water (Milli-Q^®^ Advantage A10, Merck Millipore) was used to prepare buffers and solutions.

### 3.2. Synthesis and Characterization of Compounds

The ammonium salts **2** and **3** were synthesized by the reaction of GSH or GSSG, respectively, with O,O-(–)-dimenthyl DTP **1** (Figure 1) as detailed in Supplementary Materials. The chemical structure of the compounds was confirmed using ^31^P{^1^H}, 1D ^1^H and 2D ^1^H-^13^C HSQC NMR spectroscopy on a Bruker Avance III-700 (283.42 MHz) instrument (Bruker BioSpin AG, Faellanden, Switzerland) as well as FTIR spectroscopy on a Bruker Tensor 27 spectrophotometer (Bruker BioSpin AG, Faellanden, Switzerland) in the range 400–4000 cm^–1^. The observable optical rotations α were detected on a Perkin-Elmer 341 polarimeter (Norwalk, CT, USA) at 20 °C and a concentration of c = 1 (*D*-line of sodium, λ = 589 nm, pathlength = 5 cm) and presented as specific rotations [α]^20^_D_ (grad g^−1^ cm^2^). The content of carbon, hydrogen, nitrogen and sulfur was determined on a EuroEA3000 CHNS-O analyzer (EuroVector S.p.A., Milan, Italy). The content of phosphorus was determined by the pyrolysis method (Supplementary Materials).

Hydrodynamic diameter and zeta potential were measured with the aid of the dynamic light scattering technique on a Zetasizer NanoZS analyzer (Malvern Instruments, Malvern, UK). The compounds were analyzed at a concentration of 1 mM in an aqueous 50 mM HEPES buffer (pH = 7.0).

### 3.3. Radical Scavenging Assays

DPPH assay was carried out as described previously [23]. ABTS assay was performed according to the previous procedure [44]. Radical scavenging activity of the compounds was assessed at a final concentration of 10, 100, and 1000 μM in phosphate-buffered saline (PBS, pH = 7.4). The final concentration of DPPH and ABTS probes was 0.25 mM. The incubation time was 60 and 10 min for DPPH and ABTS assays, respectively.

### 3.4. Electrochemical Analysis

The graphene oxide (GO) was suspended at a concentration of 2 mg/mL in Milli-Q water upon sonication. An aliquot of GO suspension (2 µL) was cast onto the working area of the pre-polished glassy carbon electrode (GCE, 1.5 mm in diameter) and air-dried to produce GO-modified GCE (GOE). A three-electrode system with Ag/AgCl reference electrode, nickel-based counter electrode and 0.1 M NaCl + 0.01 M sodium acetate (pH = 5) electrolyte solution were used. GOE was pretreated by means of repetitive potential scanning in the anodic region to stabilize the background signal, then rinsed with Milli-Q water and re-dried.

An aliquot of analyte solution (2 µL) in water (GSH, GSSG) or methanol (compounds **1, 2, 3**) was dropped onto GOE and air-dried. Alternatively, GOE was incubated with aqueous solutions of the compounds (500 µM in PBS) for 5 min and then rinsed with Milli-Q water. The adsorbed species were analyzed using the square-wave voltammetry technique in the potential range from 0 to 1.1 V at a frequency of 10 Hz, an amplitude of 10 mV and a potential step of 5 mV. The measurements were performed on an EmStat potentiostat (PalmSens, Houten, Netherlands). The voltammograms were treated with PSTrace 5.5 software (Analytical mode).

### 3.5. Ellman Assay

The stock solution of DTNB was prepared at a concentration of 10 mM in DMSO. GSH, DTP and their derivatives were dissolved at a concentration of 1 mM in PBS with 8 mg/mL Triton X100 in a tube covered with gas-permeable film. The mixture was incubated for 56 h at room temperature upon constant stirring, and aliquots of air-oxidized products were collected every 2–8 h. For thiol group determination, the aliquots were mixed with DTNB at a final concentration of the compounds of 0.5 mM followed by 2-min incubation and registration of the optical absorbance of the resultant solution at λ = 412 nm on an Infinite 200 PRO microplate analyzer (Tecan, Männedorf, Switzerland).

### 3.6. Probing with MTS Indicator

The effector compounds were mixed with MTS tetrazolium salt in Triton X100 supplemented PBS at concentrations of 0.1–10 mM (compound) and 0.5 mM (MTS). The mixture was incubated for 2 h at room temperature, and the optical absorbance of reduced MTS (formazan) was detected every 10 min at λ = 490 nm on an Infinite 200 PRO microplate analyzer. SOD-2 (500 U/mL) was used to eliminate the superoxide radical generated during the reaction.

### 3.7. NMR Analysis

1D (^1^H, ^31^P{^1^H}) and 2D ^1^H-^13^C NMR spectra of the compounds in aqueous solutions were registered on a 700 MHz NMR spectrometer (AVANCE III-700, BrukerBiospin AG, Fällanden, Switzerland) equipped with a quadruple resonance (^1^H, ^13^C, ^15^N, and ^31^P) CryoProbe at 298 K. The spectrometer operated in the mode of internal stabilization of the resonance line ^2^H. ^1^H NMR spectra were recorded using 90° pulses, a relaxation delay of 2 s and a spectral width of 12.00 ppm.

To assign ^1^H signals spin-spin constants, signal multiplicity and integral intensities from ^1^H NMR spectra were used. To assign ^13^C signals, the HSQC pulse sequence was applied. For each HSQC spectrum, 128 transients were collected using two dummy scans with spectral widths of 12 ppm in F2 and 200 ppm in F1 dimensions, using 4096 complex points for F2 and 512 complex points for F1. Chemical shifts were measured relative to 4,4-dimethyl-4- silapentan-1-sulfonic acid (DSS). Spectra were processed using Bruker software TOPSPIN (v. 3.6). The data were Fourier transformed with a sine-bell-squared window function shifted between π/2 and π/4. A polynomial baseline correction was applied to both sides of the residual water signal.

### 3.8. GC–MS Analysis

The compounds **1**, **2**, **3** (0.5 g, 0.7 mmol) were dissolved in 10 mL of anhydrous methanol in a hermetic vial. Distilled water (1 mL) was added dropwise to the solution at room temperature, followed by vial closure. The gas chromatography–mass spectrometry (GC–MS) analysis was performed on a TRACE 1310 gas chromatograph coupled with an ISQ (ThermoFisher Scientific, Bremen, Germany) quadrupole mass-spectrometric detector and a TriPlus RSH autosampler (CTC Analytica AG, Brescia, Italy). High-purity helium was used as a carrier gas at a constant flow rate of 15 mL/min. The samples were injected with a gas-tight syringe at a temperature of 250 °C with a split flow of 1:2. Gas separation was carried out on an Agilent PoraBOND Q (Agilent Technologies, CA, USA) capillary column (50 m × 0.53 mm × 10.0 μm). The temperature regime was initially kept at 40 °C for 7 min, then increased to 135 °C at 125 °C /min and kept for 4 min, and further increased to 300 °C at 125 °C /min and kept for 1.5 min. The total run time was 14.5 min. Scan parameters were set to measure *m*/*z* in the range from 14 to 50 (air—29 *m*/*z*; carbon dioxide—44 *m*/*z*; water—18 *m*/*z*; hydrogen sulfide—34 m/z; methyl alcohol—32 *m*/*z*; ethanol—46 *m*/*z*).

(–)-(1R,2S,5R)-menthol was analyzed using a DFS Thermo Electron Corporation mass spectrometer at an ionization potential of 70 eV and Thermo TG-5MS capillary column (30 m × 0.25 mm) (ThermoFisher Scientific, Bremen, Germany). Chromatographic parameters were as follows: injection temperature 280 °C, split flow 1:10, initial temperature 60 °C (1 min), heating at 10 °C/min to 120 °C (1 min), heating at 20 °C/min to 280 °C (20 min), gas flow rate 2.9 mL/min. Data acquisition and processing were performed using Thermo Xcalibur software and the NIST-14 database.

## 4. Conclusions

Our results identify the ammonium salts of glutathione and O,O-(–)-dimenthyl DTP as a dynamic redox-active system with improved antioxidant and electrochemical properties over unmodified tripeptide. These salts can be considered as novel bioactive amphiphilic derivatives of glutathione, which generate redox modulating and inter-converting intermediates such as H_2_S, disulfide and thiols as well as other natural products (phosphoric acid, menthol). Recently, the GSH dithiophosphates with different O,O-diorganyl groups were shown to possess increased anticancer activity in vitro, whereas they did not stimulate the proliferation of cancer cells, unlike free GSH [23]. The structure and activity of DTP moiety in glutathione dithiophosphates can be tailored by introducing different O,O-groups with required properties.

## Data Availability

The data presented in this study are available on request from the corresponding author.

## Supplementary Materials

Synthesis of glutathione dithiophosphates, Figure S1: 1D ^31^P{^1^H} NMR spectrum of GSH-DTP in H_2_O–D_2_O, Figure S2: 1D ^31^P{^1^H} NMR spectrum of GSH-DTP in H_2_O–D_2_O + DPC, Figure S3: 1D ^31^P{^1^H} NMR spectrum of GSH-DTP in PBS–D_2_O, Figure S4: 1D ^31^P{^1^H} NMR spectrum of GSH-DTP in BBS–D_2_O, Figure S5: 1D ^31^P{^1^H} NMR spectrum of GSSG-(DTP)_2_ in BBS–D_2_O, Figure S6: 2D ^1^H-^13^C HSQC NMR spectrum of GSSG in H_2_O–D_2_O, Figure S7: 2D ^1^H-^13^C HSQC NMR spectrum of GSH in H_2_O–D_2_O, Figure S8: Fragments of 1D ^1^H NMR spectra of GSSG and GSH in BBS–D_2_O, Figure S9: 2D ^1^H-^13^C HSQC NMR spectrum of GSH-DTP in BBS–D_2_O, Figure S10: 2D ^1^H-^13^C HSQC NMR spectrum of GSSG-(DTP)_2_ in BBS–D_2_O.

## Sample Availability

Samples of the compounds are not available from the authors.
