# Supplementary material for: Dithiophosphate-Induced Redox Conversions of Reduced and Oxidized Glutathione
Source: Molecules. 2021 May 17;26(10):2973. doi: 10.3390/molecules26102973 (PMC8157023; doi:10.3390/molecules26102973)
Supplement: Supplementary file 1 [file molecules-26-02973-s001.zip › molecules-1179693-supplementary.pdf]

Supplementary Materials

Dithiophosphate-Induced Redox Conversions of Reduced and Oxidized Glutathione

Rezeda A. Ishkaeva [1], Ilyas S. Nizamov [2,5], Dmitriy S. Blokhin [1,3], Elizaveta A. Urakova [3], Vladimir V. Klochkov [3], Ilnar D. Nizamov [2], Bulat I. Gareev [4], Diana V. Salakhieva [1] and Timur I. Abdullin [1,*]

1 Department of Biochemistry, Biotechnology, and Pharmacology, Institute of Fundamental Medicine and Biology, Kazan Federal University, 420008 Kazan, Russia; RezAAhmadishina@kpfu.ru (R.A.I.); Dmitrij.Blohin@kpfu.ru (D.S.B.); DiVitSai@gmail.com (D.V.S.)
2 Department of High Molecular and Organoelement Compounds, Alexander Butlerov Institute of Chemistry, Kazan Federal University, 420008 Kazan, Russia; Ilyas.Nizamov@kpfu.ru (I.S.N.); IDNizamov@kpfu.ru (I.D.N.)
3 Department of Medical Physics, Institute of Physics, Kazan Federal University, 420008 Kazan, Russia; urakova.li@yandex.ru (E.A.U.); Vladimir.Klochkov@kpfu.ru (V.V.K.)
4 Laboratory of Isotope and Element Analysis, Institute of Geology and Petroleum Technologies, Kazan Federal University, 420008 Kazan, Russia; bulat@gareev.net
5 Laboratory of Organometallic and Coordination Compounds, A.E. Arbuzov Institute of Organic and Physical Chemistry, Kazan Scientific Center, Russian Academy of Sciences, 420088 Kazan, Russia
* Correspondence: timur.abdullin@kpfu.ru or tabdulli@gmail.com; Tel.: +7-(843)-236-7640

Citation: Ishkaeva, R.A.; Nizamov, I.S.; Blokhin, D.S.; Urakova, E.A.; Klochkov, V.V.; Nizamov, I.D.; Gareev, B.I.; Salakhieva, D.V.; Abdullin, T.I. Dithiophosphate-Induced Redox Conversions of Reduced and Oxidized Glutathione. *Molecules* **2021**, *24*, 2973. https://doi.org/10.3390/molecules26102973

Academic Editor: Fintan Kelleher
Received: 26 March 2021
Accepted: 14 May 2021
Published: 17 May 2021

Publisher's Note: MDPI stays neutral with regard to jurisdictional claims in published maps and institutional affiliations.

Copyright: © 2021 by the authors. Licensee MDPI, Basel, Switzerland. This article is an open access article distributed under the terms and conditions of the Creative Commons Attribution (CC BY) license (http://creativecommons.org/licenses/by/4.0/).

Reduced glutathione salt of O,O-bis[(–)-(1R,2S,5R)-2-iso-propyl-5-methylcyclohex-1-yl] dithiophosphoric acid (2):

Dithiophosphoric acid **1** (0.26, 0.64 mmol) was added dropwise to the solution of reduced glutathione (0.2 g, 0.65 mmol) in 10 mL anhydrous ethanol under dry argon bubbling and stirring at 20°C; the reaction was continued for 2.5 h at 50°C. The mixture was filtered, and the filtrate was evaporated at reduced pressure (0.5 mm Hg) at 40°C for 1 h and then in vacuum (0.02 mm Hg) to give salt **2** (0.4 g, 87%) as a white solid, mp 78-80°C, $[\alpha]^{20}_D$ +4.4 grad.g^{-1}.cm^2 (c = 0.99, ethanol). FTIR (cm^{-1}, film): ν_{max} 3355 s (NH_3^+), 2955 st, 2930 st, 2870 st $\nu_{as,s}$(CH_3), $\nu_{as,s}$(CH_2), 2600 w (S–H), 1730 st (O=C–O), 1653 st (NHC=O, Amide I), 1538 st (CNH, Amide II), 1454 m δ_{as} (CH_3), 1387 m, 1370 m δ_s((CH_3)$_2$C *gem*), 1044 st ((P)O–C), 959 st (OC–C, C–C), 670 st (P=S), 550 m (P–S). ^{31}P–{^{1}H} NMR (ethanol) ppm: δ = 105.8. Anal. Calcd. for $C_{30}H_{56}N_3O_8PS_3$: C, 50.47; H, 7.91; N, 5.89; P, 4.34; S, 13.47%. Found: C, 50.76; H, 8.23; N, 6.16; P, 4.65; S, 13.76%.

Oxidazed glutathione salt of O,O-bis[(–)-(1R,2S,5R)-2-iso-propyl-5-methylcyclohex-1-yl] dithiophosphoric acid (3):

Dithiophosphoric acid **1** (0.4, 0.98 mmol) was added dropwise to the solution of oxidized glutathione (0.3 g, 0.49 mmol) in 10 mL anhydrous ethanol under dry argon bubbling and stirring at 20°C; the reaction was continued for 1.5 h at 50°C. The mixture was filtered, and the filtrate was evaporated at reduced pressure (0.5 mm Hg) at 40°C for 1 h and then in vacuum (0.02 mm Hg) to give salt **3** (0.6 g, 86%) as a semisolid, $[\alpha]^{20}_D$ –3.7 grad.g^{-1}.cm^2 (c = 0.93, ethanol). FTIR (cm^{-1}, film): ν_{max} 3298 m br (NH_3^+), 2956 st, 2927 st, 2870 s $\nu_{as,s}$(CH_3), $\nu_{as,s}$(CH_2), 2600 w (S–H), 1731 st (O=C–O), 1658 st (NHC=O, Amide I), 1538 m (CNH, Amide II), 1456 m δ_{as} (CH_3), 1387 m, 1371 m δ_s((CH_3)$_2$C *gem*), 1045 st, 1022 st ((P)O–C), 976 st, 964 st (OC–C, C–C), 672 st (P=S), 576 m (P–S). ^{31}P–{^{1}H} NMR (ethanol) ppm: δ = 109.3. Anal. Calcd. for $C_{60}H_{110}N_6O_{16}P_2S_6$: C, 50.54; H, 7.78; N, 5.89; P, 4.34; S 13.49%. Found: C, 50.18; H, 8.12.; N, 5.56; P, 4.02; S 13.86%.

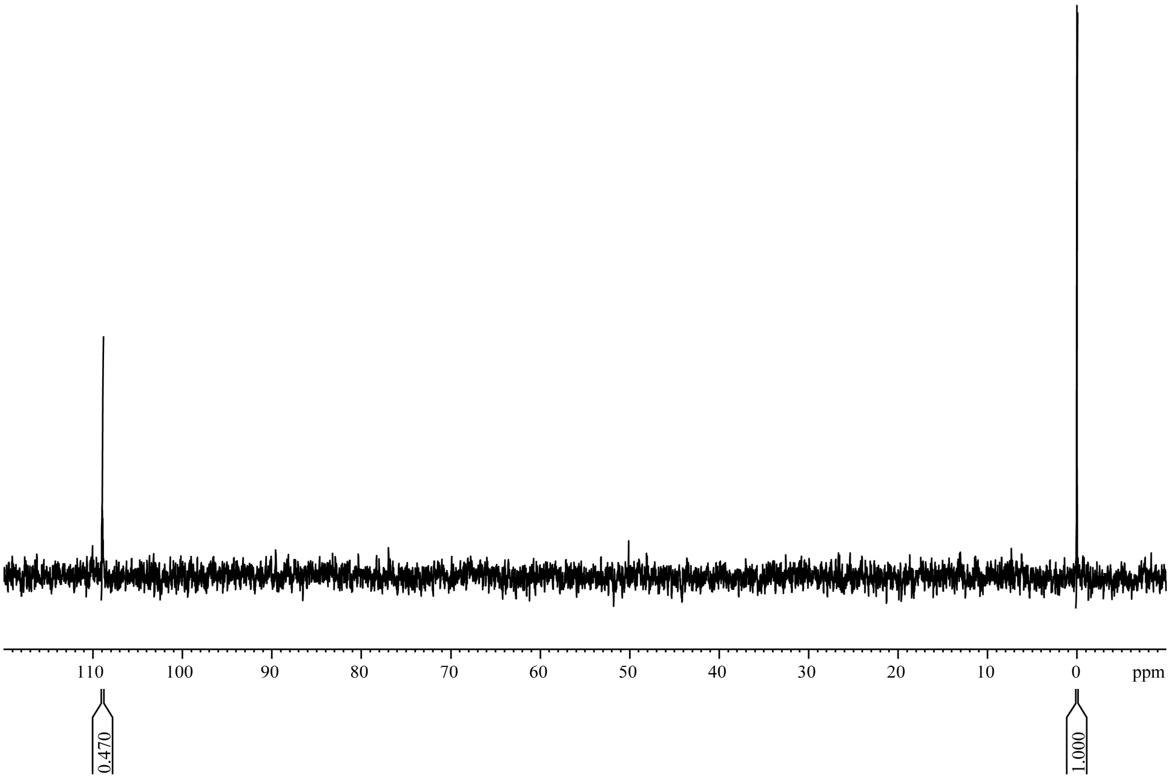

Figure S1. 1D $^{31}P\{^{1}H\}$ NMR spectrum of GSH-DTP in the H_2O–D_2O system at 25 ^{o}C.

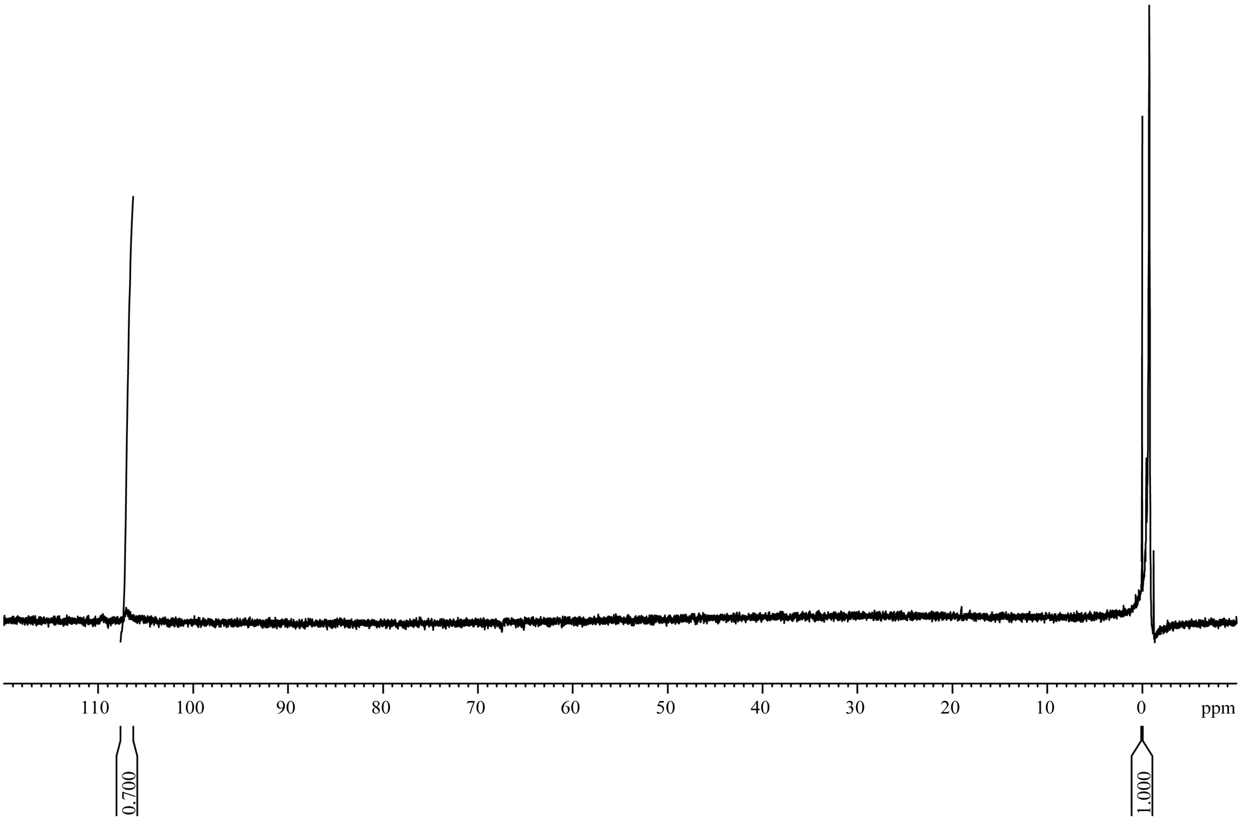

Figure S2. 1D $^{31}P\{^{1}H\}$ NMR spectrum of GSH-DTP in the H_2O–D_2O system supplemented with DPC at 25 ^{o}C.

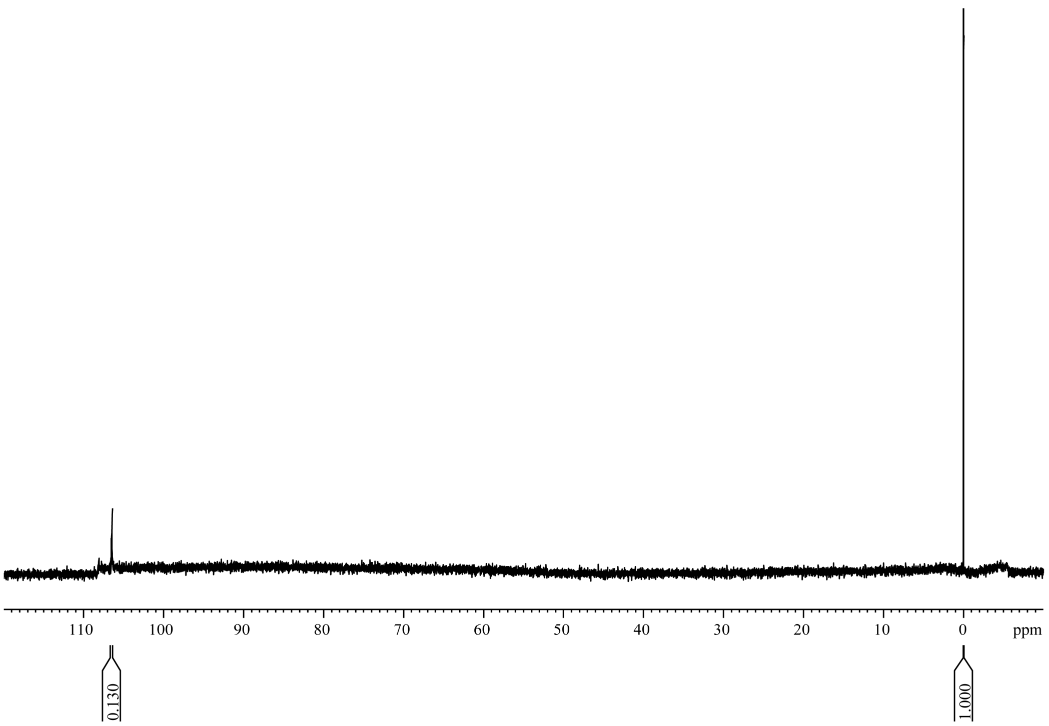

Figure S3. 1D $^{31}P\{^{1}H\}$ NMR spectrum of GSH-DTP in the PBS–D_2O system at 25 °C (pH = 7.4).

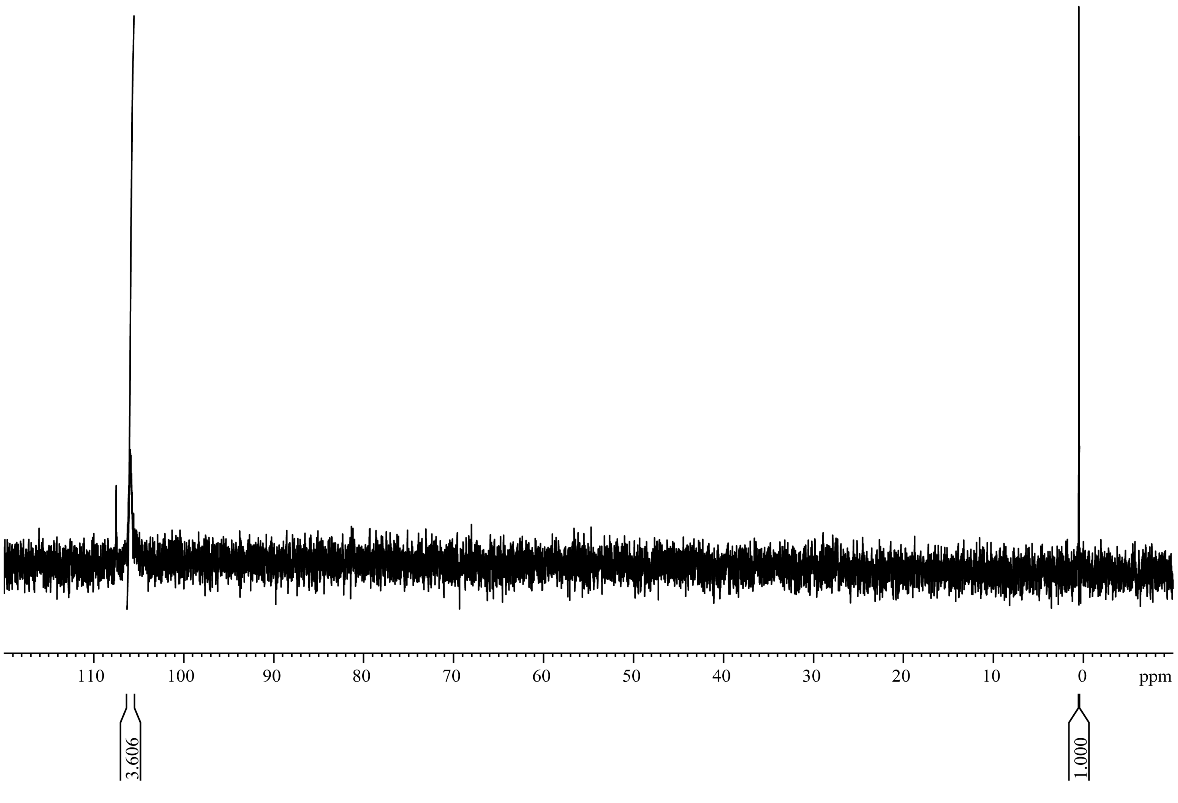

Figure S4. 1D $^{31}P\{^{1}H\}$ NMR spectrum of GSH-DTP in the BBS–D_2O system at 25 °C (pH = 7.4).

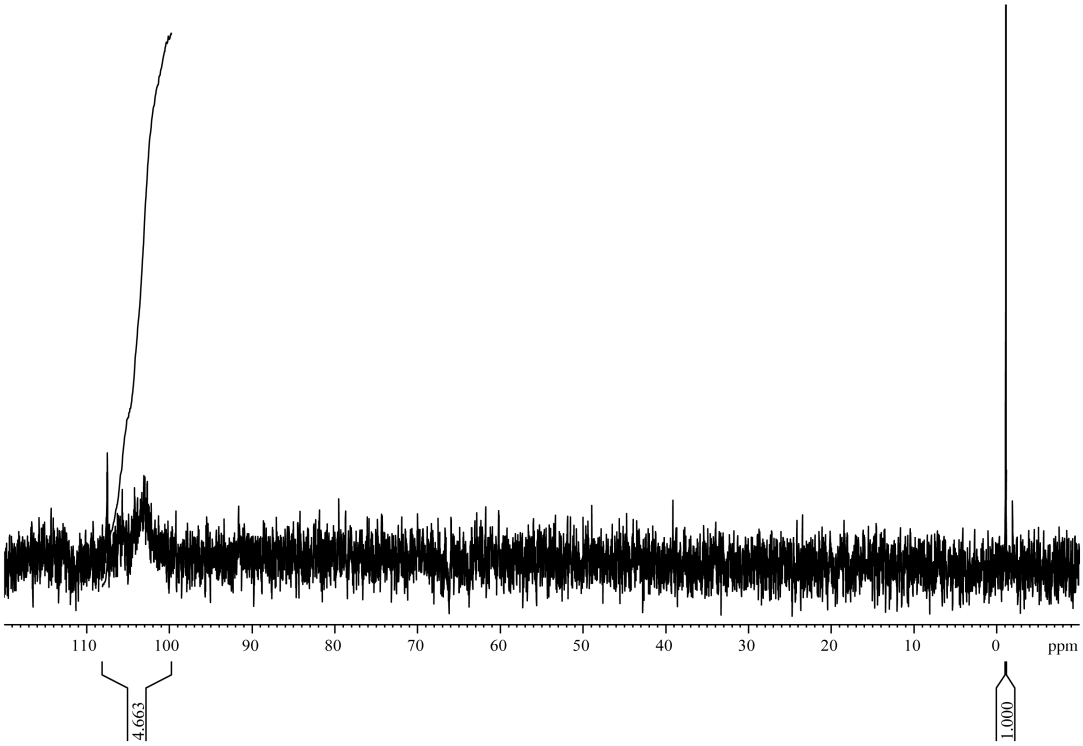

Figure S5. 1D $^{31}P\{^{1}H\}$ NMR spectrum of GSSG-(DTP)$_2$ in the BBS–D$_2$O system at 25 ^{o}C (pH = 7.4).

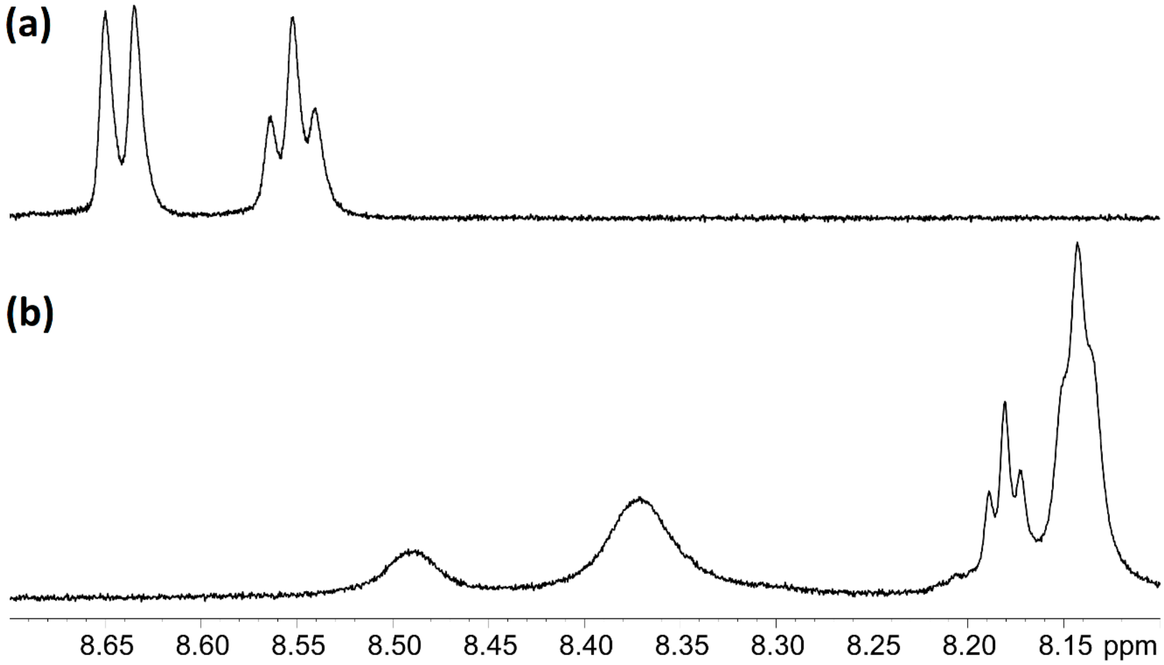

Figure S6. Fragments of 1D ^{1}H NMR spectra of GSSG (a) and GSSG-(DTP)$_2$ (b) at 25 °C in the BBS–D$_2$O system (pH = 7.4).

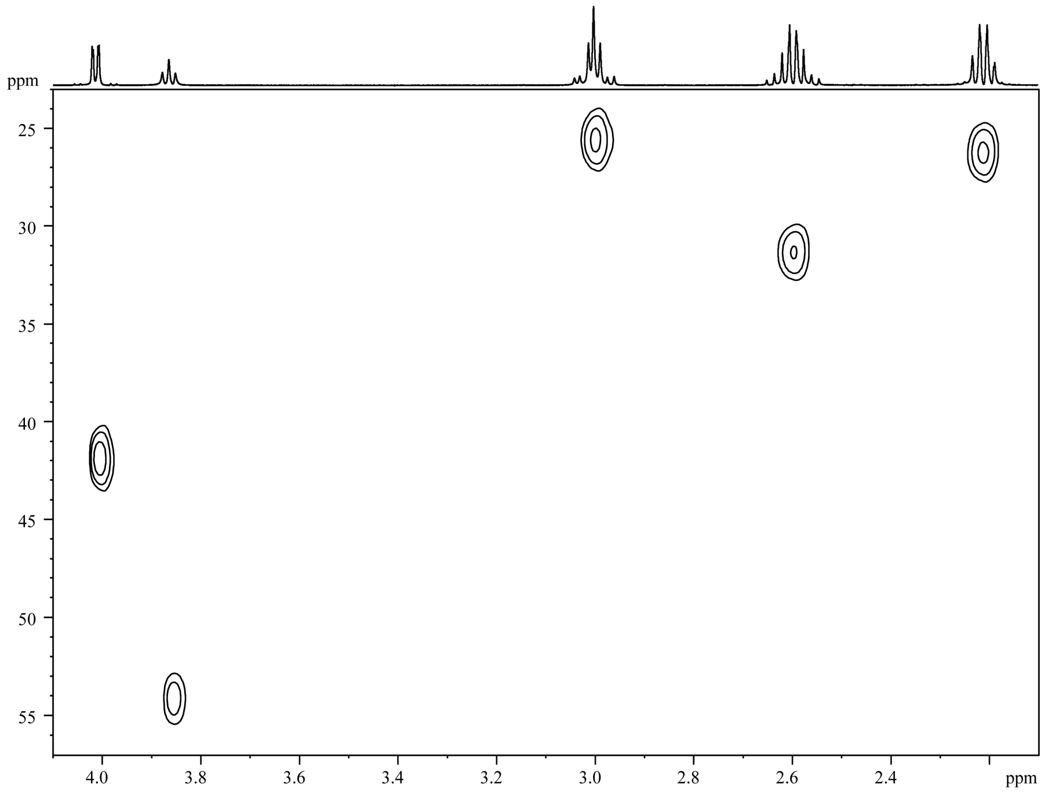

Figure S7. 2D ^{1}H-^{13}C HSQC NMR spectrum of GSH in the H_2O–D_2O system at 25 °C.

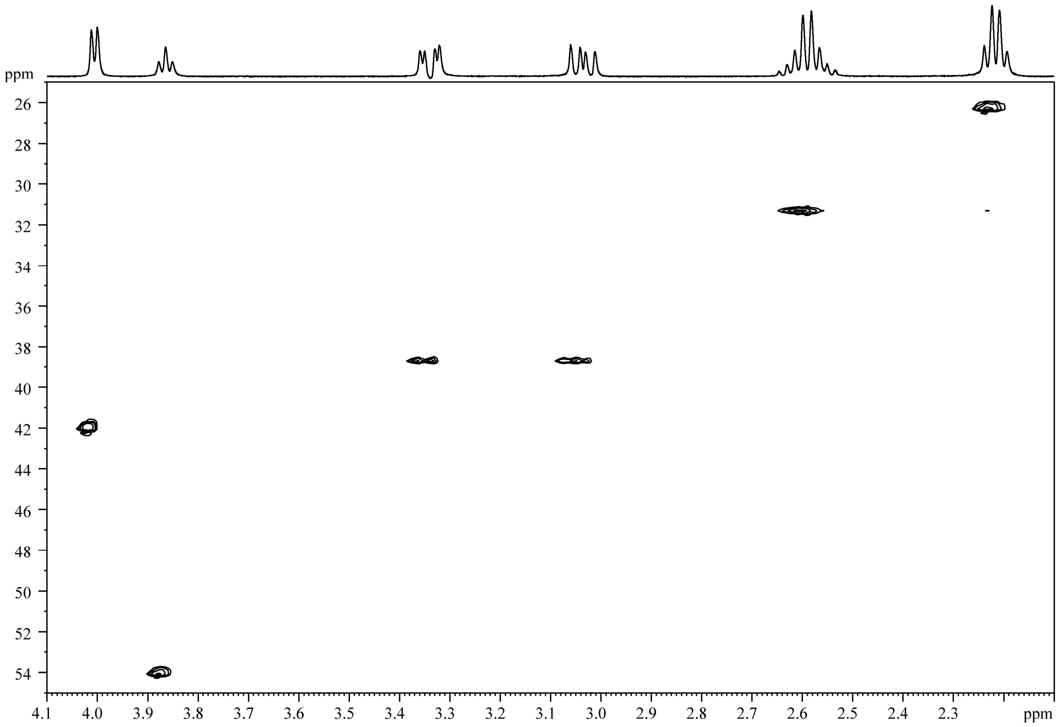

Figure S8. 2D ^{1}H-^{13}C HSQC NMR spectrum of GSSG in the H_2O–D_2O system at 25 °C.

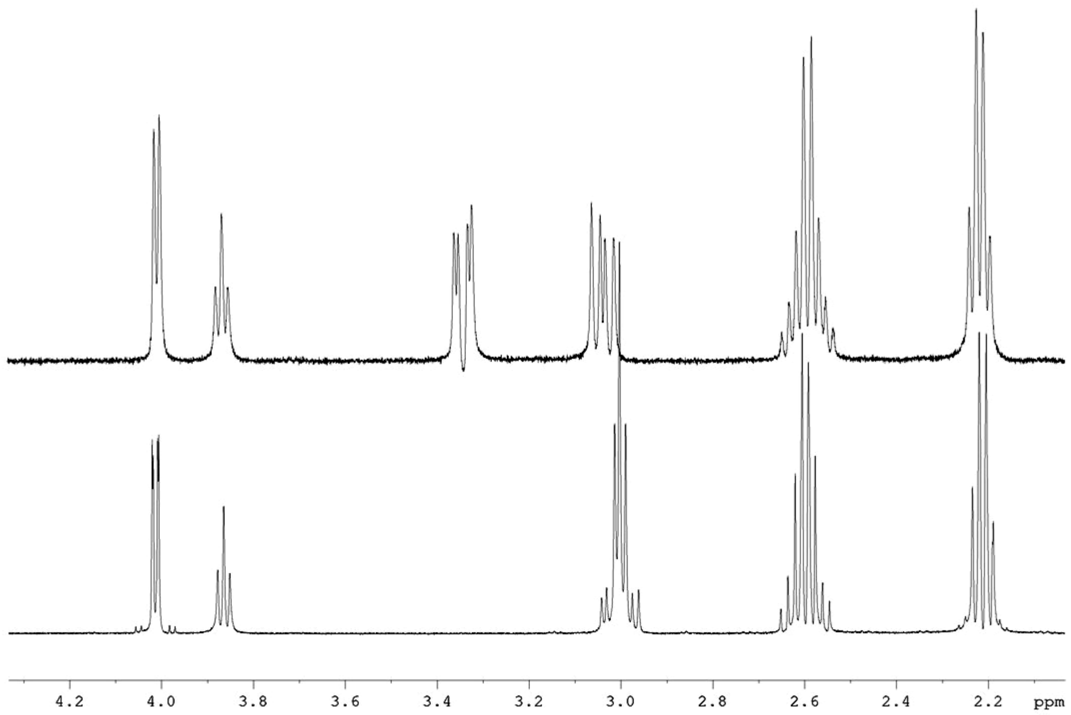

Figure S9. Fragments of 1D ^{1}H NMR spectra of GSSG (upper spectrum) and GSH (lower spectrum) in the BBS–D_2O system. Glu Hγ protons of both GSH and GSSG gave a signal at 2.2 ppm. Two multiplets at 2.6 ppm in both spectra were assigned to Glu Hβ protons. Cys Hβ protons of GSH provided two doublets at 3.0 ppm, whereas analogous signals in GSSG were split into two pairs of doublets at 3.01 and 3.36 ppm. Triplet at 3.85 ppm was attributed to Gly Hα proton of both GSH and GSSG. Glu Hα protons resonated as two singlets at 4.0 and 4.01 ppm in both spectra.

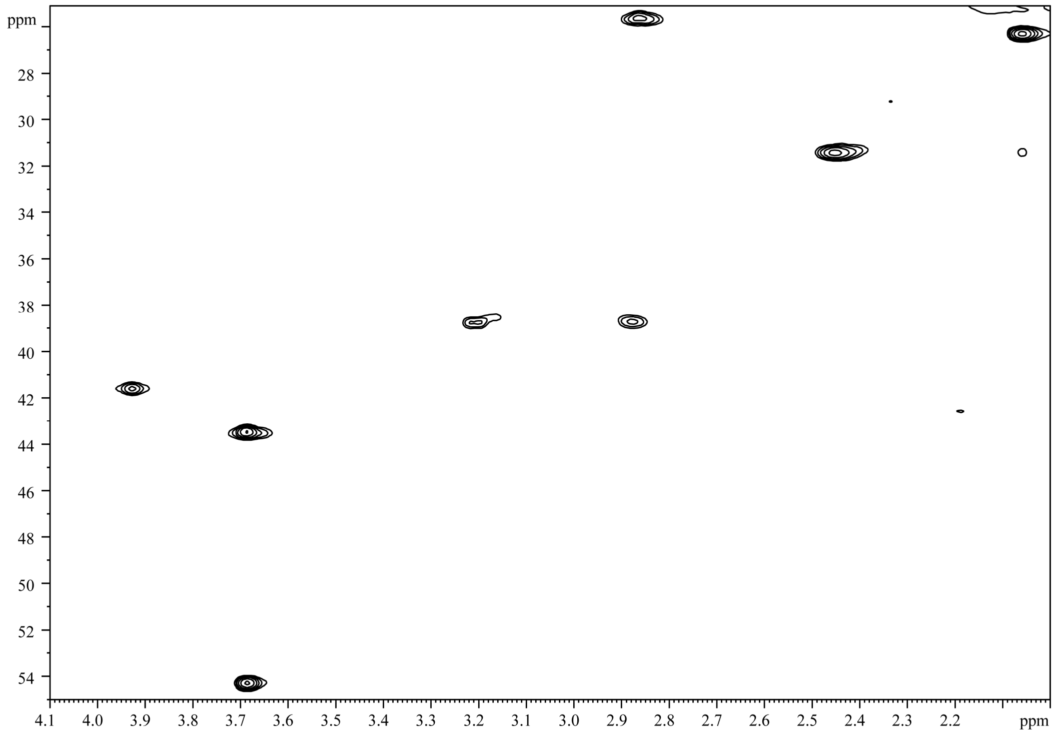

Figure S10. 2D ^{1}H-^{13}C HSQC NMR spectrum of GSSG-$(DTP)_2$ in the BBS–D_2O system at 25 °C (pH=7.4).

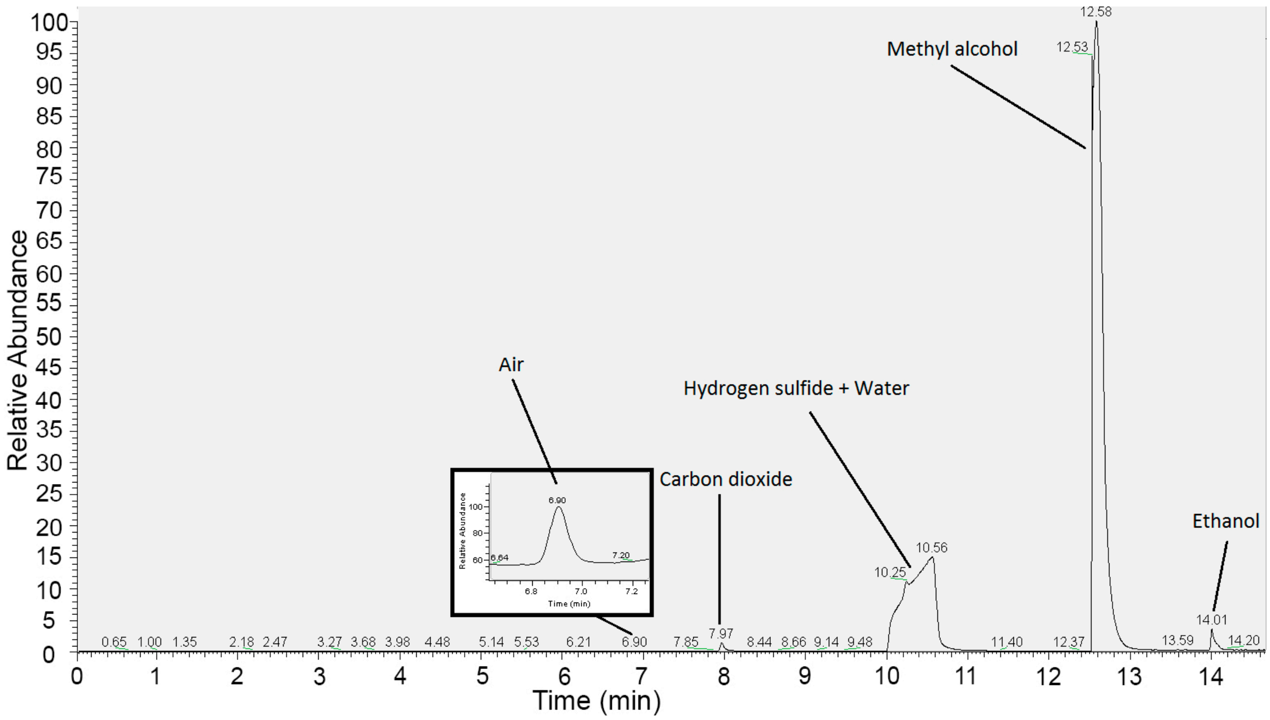

Figure S11. GC–MS analysis of hydrolysis products of GSH-DTP in a methanol–water mixed solution (liquid phase).

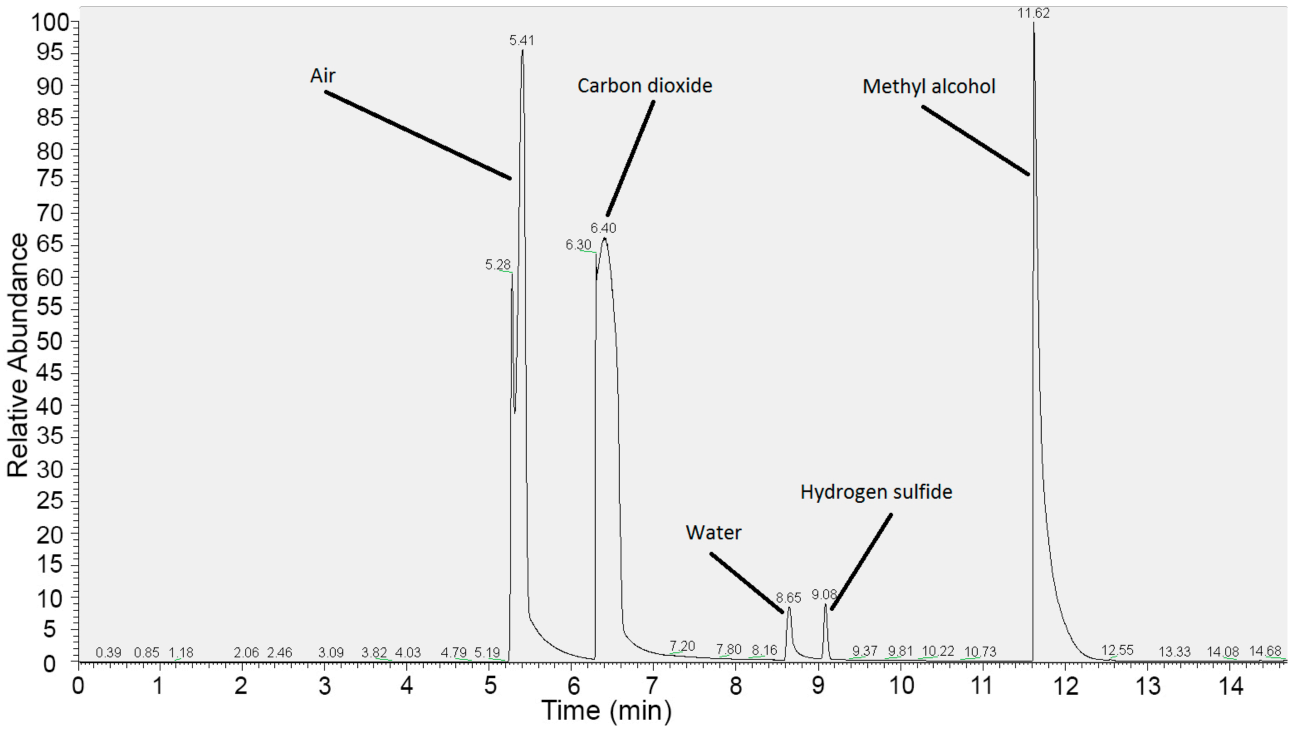

Figure S12. GC–MS analysis of hydrolysis products of DTP in a methanol–water mixed solution (gas phase).

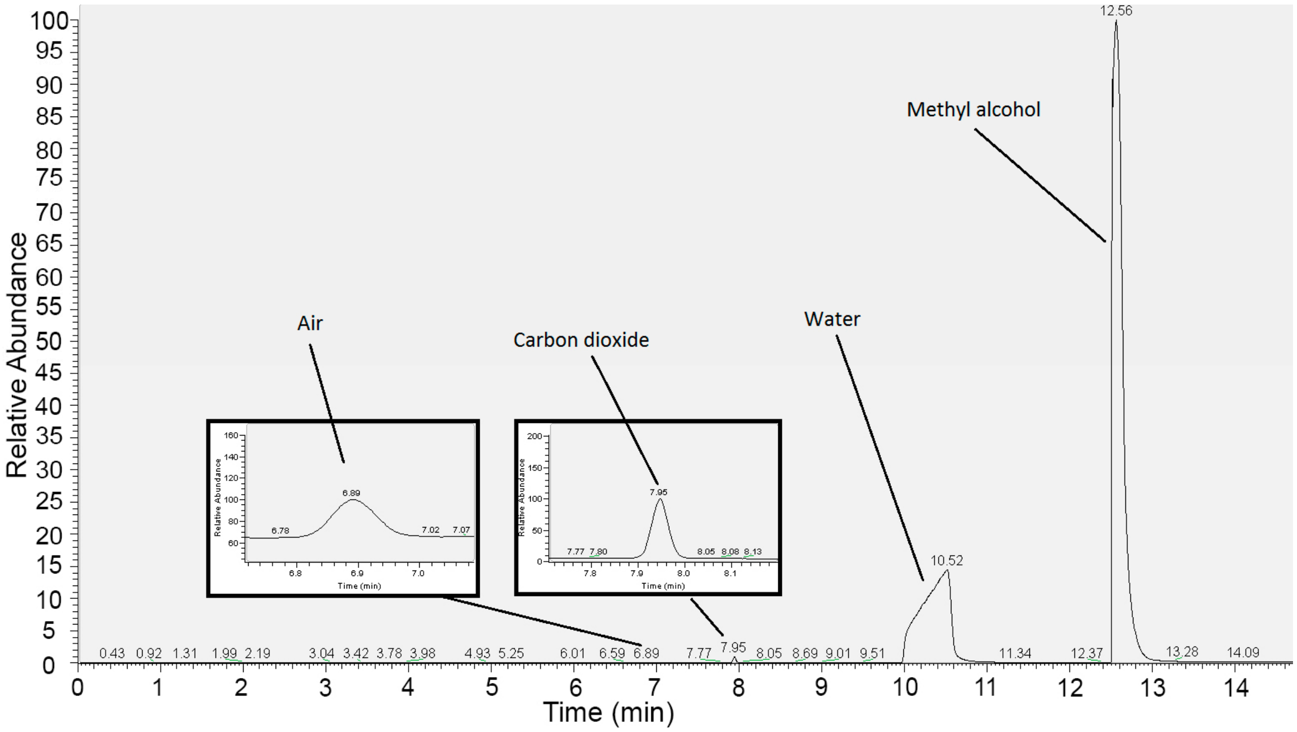

Figure S13. GC–MS analysis of hydrolysis products of DTP in a methanol–water mixed solution (liquid phase).

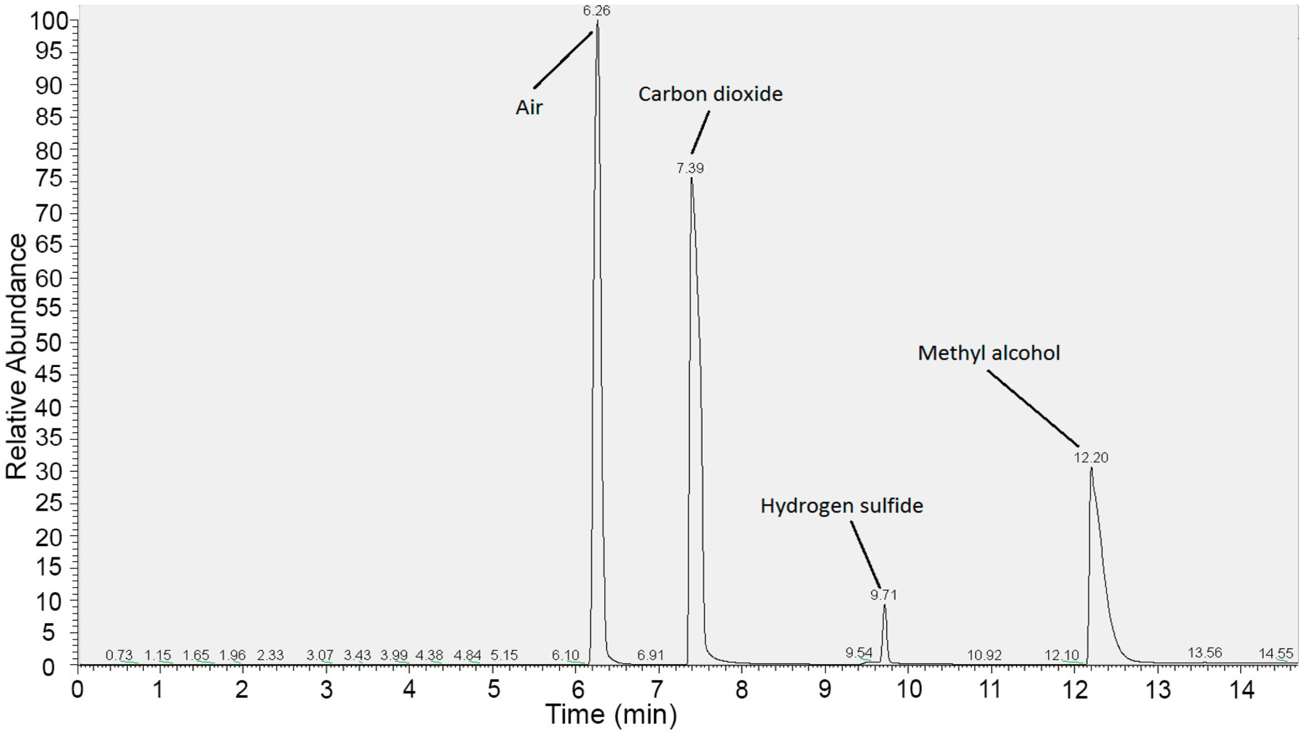

Figure S14. GC–MS analysis of hydrolysis products of GSSG-$(DTP)_2$ in a methanol–water mixed solution (gas phase).

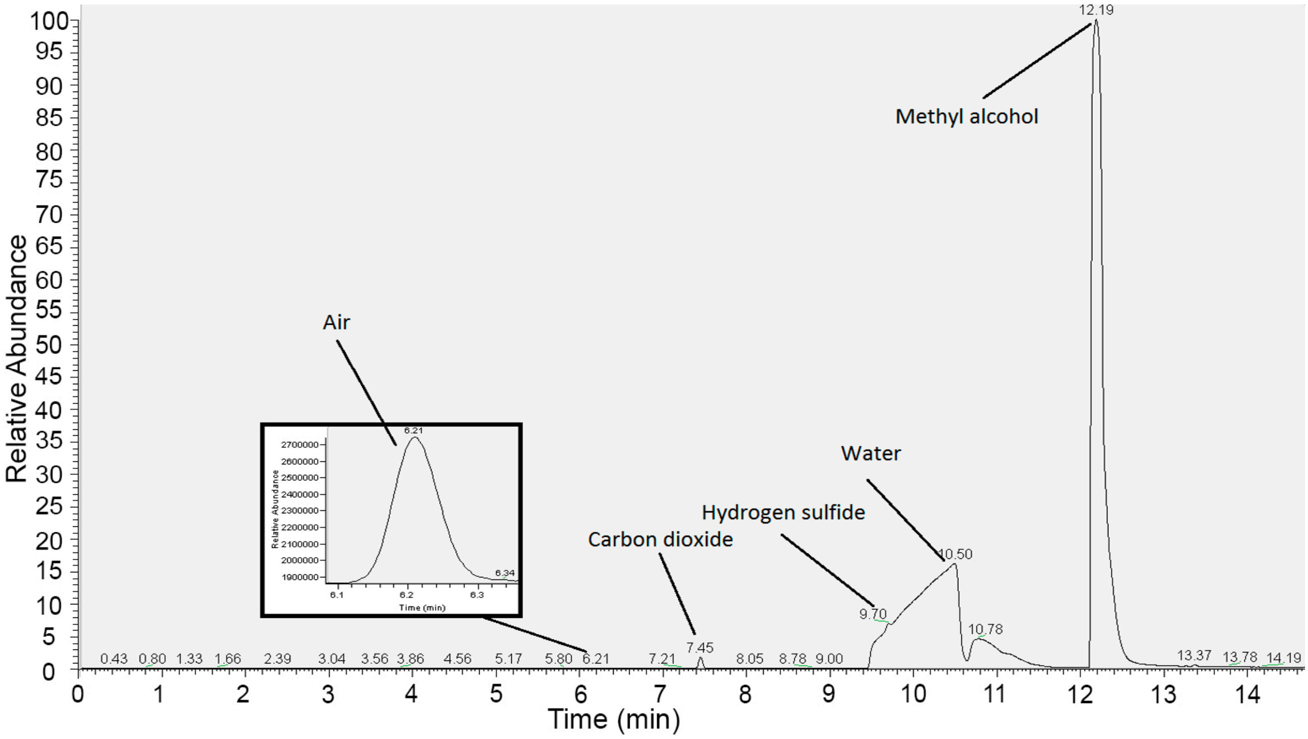

Figure S15. GC–MS analysis of hydrolysis products of GSSG-$(DTP)_2$ in a methanol–water mixed solution (liquid phase).
